# Dissecting the Genetic Basis for Seed Coat Mucilage Heteroxylan Biosynthesis in *Plantago ovata* Using Gamma Irradiation and Infrared Spectroscopy

**DOI:** 10.3389/fpls.2017.00326

**Published:** 2017-03-21

**Authors:** Matthew R. Tucker, Chao Ma, Jana Phan, Kylie Neumann, Neil J. Shirley, Michael G. Hahn, Daniel Cozzolino, Rachel A. Burton

**Affiliations:** ^1^Australian Research Council Centre of Excellence in Plant Cell Walls, University of Adelaide, Waite Campus, Urrbrae, SAAustralia; ^2^School of Agriculture, Food and Wine, University of Adelaide, Waite Campus, Urrbrae, SAAustralia; ^3^Complex Carbohydrate Research Center, University of Georgia, Athens, GAUSA

**Keywords:** *Plantago*, mucilage, xylan, spectroscopy, seed, mutagenesis

## Abstract

Seeds from the myxospermous species *Plantago ovata* release a polysaccharide-rich mucilage upon contact with water. This seed coat derived mucilage is composed predominantly of heteroxylan (HX) and is utilized as a gluten-free dietary fiber supplement to promote human colorectal health. In this study, a gamma-irradiated *P. ovata* population was generated and screened using histological stains and Fourier Transform Mid Infrared (FTMIR) spectroscopy to identify putative mutants showing defects in seed coat mucilage HX composition and/or structure. FTMIR analysis of dry seed revealed variation in regions of the IR spectra previously linked to xylan structure in *Secale cereale* (rye). Subsequent absorbance ratio and PCA multivariate analysis identified 22 putative mutant families with differences in the HX IR fingerprint region. Many of these showed distinct changes in the amount and subtle changes in structure of HX after mucilage extrusion, while 20% of the putative HX mutants identified by FTMIR showed no difference in staining patterns of extruded mucilage compared to wild-type. Transcriptional screening analysis of two putative *reduced xylan in mucilage* (*rxm*) mutants, *rxm1* and *rxm3*, revealed that changes in HX levels in *rxm1* correlate with reduced transcription of known and novel genes associated with xylan synthesis, possibly indicative of specific co-regulatory units within the xylan biosynthetic pathway. These results confirm that FTMIR is a suitable method for identifying putative mutants with altered mucilage HX composition in *P. ovata*, and therefore forms a resource to identify novel genes involved in xylan biosynthesis.

## Introduction

Xylans are polysaccharides found in plant cell walls and are almost as ubiquitous as cellulose. Apart from prominent economic value in the livestock feed and pharmaceutical industries, they are also an important source of dietary fiber for human consumption (Cummings et al., 1992; Gunness and Gidley, 2010). The backbone of xylan is typically comprised of 1→4 linked xylosyl residues, and distinct physicochemical properties are conferred by substitutions and side chains composed of sugars such as arabinose, xylose or glucuronic acid (Ebringerová, 2006). Considerable effort has been directed toward understanding the mechanism of xylan biosynthesis (York and O’Neill, 2008; Faik, 2010; Rennie and Scheller, 2014), with studies being undertaken in various species including *Arabidopsis*, rice, poplar, wheat and *Plantago* (Anders et al., 2012; Chiniquy et al., 2012; Haughn and Western, 2012; Jensen et al., 2013; Lovegrove et al., 2013; Phan et al., 2016). These studies show that multiple genes, particularly those from diverse glycosyltransferase (GT) families, are involved in xylan synthesis and substitution. Despite this, dissection of the xylan biosynthetic machinery, the interaction between different biosynthetic components and the identification of upstream regulators has proved challenging to dissect in traditional dicot systems where xylan is predominately deposited in thickened, vascular cell walls. Only recently has the *Arabidopsis thaliana* seed coat mucilage been revealed as an alternative model to study xylan biosynthesis, which may provide new opportunities to investigate key regulatory and biochemical activities (Voiniciuc et al., 2015; Hu et al., 2016; Ralet et al., 2016).

Another model for the study of heteroxylan (HX) biosynthesis is *Plantago ovata* (psyllium), an annual herb that produces a polysaccharide-rich seed coat mucilage (Jensen et al., 2013, 2014; Phan et al., 2016). Similar to other myxospermous plants such as *Linum usitatissimum* (flax) and *Arabidopsis*, mucilage is extruded from the seed coat upon hydration to form a viscous water-trapping gel around the seed, providing a moist environment for germination and growth (Western, 2012). In contrast to *Arabidopsis*, *P. ovata* mucilage contains small amounts of pectin and cellulose, and large amounts (~90%) of HX (Fischer et al., 2004; Phan et al., 2016). HX is synthesized in the seed coat from 7 days after pollination (Phan et al., 2016) and contains unusual substitutions compared to other xylan types, such as single xylose units appended to the backbone and Ara*f*-α-(1→3)-Xyl*p*-β-(1→3)-Ara*f* trisaccharide branches. These structures are likely synthesized by enzymes from the glycosyl transferase 61 (GT61) family (Phan et al., 2016), thereby influencing HX solubility and thus the behavior of the mucilage as a dietary fiber. Study of the synthesis of HX structure in *P. ovata* can therefore lead to an understanding of xylan synthesis in general, providing tools for the manipulation of dietary fiber for humans.

One approach to study the HX biosynthetic pathway and gene regulation in *P. ovata* is through the analysis of mutants. Previous studies have reported that gamma irradiation is an effective mutagen in *P. ovata* that can be used to induce mutations influencing flowering time, growth habit and disease resistance (Dhar et al., 2005). Despite this, a large-scale genetic screen for changes in mucilage synthesis has yet to be reported. The challenge of such an approach is that a sequenced genome is currently unavailable for *P. ovata*, and different accessions show a remarkable lack of diversity required for traditional map-based cloning (Dhar et al., 2009). However, these limitations can potentially be overcome through transcriptome sequencing, segregant analysis, and genotyping-by-sequencing approaches. An additional challenge is the screening process, which typically requires large amounts of seed, destructive analyses, time and labor. Conventional screening methods for mucilage mutants have used diagnostic stains, such as ruthenium red (RR), to target phenotypic differences compared to wild-type (WT). A complementary technique that offers promise for rapid, non-destructive screening is Infrared (IR) spectroscopy. IR spectroscopy is a classical method used to analyze molecular composition based on detection of particular chemical bonds in complex molecules (Barth, 2007). In the past two decades, IR has shown potential in a large range of applications, from compositional determination of proteins, lipids and carbohydrates, to detection of substrates containing subtle chemical differences (Cozzolino et al., 2015). A successful example is the application of Fourier Transform Infrared (FT-IR) microspectroscopy to identify different classes of *Arabidopsis* cell wall mutants based on cellulose defects (Mouille et al., 2003).

Infrared spectroscopy methods have been used to examine characteristics of xylan polysaccharides since the early 1990s. Kačuráková et al. (1994) used IR techniques to study purified rye bran arabinoxylan (AX) fractions, and related peak intensity ratios to differences in xylan structure. Their findings provided a semi-quantitative evaluation of AX, related to position *O-3* substitution. A linear relationship between decreasing intensity ratios and increasing arabinose content was observed. Based on the structural similarity of *O-3* substitutions in rye bran AX and *P. ovata* HX (Fischer et al., 2004; Saghir et al., 2008; Phan et al., 2016), we hypothesized that these ratios might allow discrimination of *P. ovata* HX structures containing varying substitution levels. This study therefore aimed to use Fourier Transform Mid-Infrared spectroscopy (FTMIR) techniques to screen for compositional variation in HX within a *P. ovata* mutant population.

The results presented here show that FTMIR techniques can identify putative *P. ovata* mucilage mutants based on the ratios previously used for rye AX. Distinctive phenotypic differences in seed mucilage were observed in ~80% of the putative FTMIR mutants when tested with conventional RR staining methods. Chemical analysis indicated that several different classes of putative mutants were identified; (i) heavily reduced xylan, (ii) partially reduced xylan, (iii) increased xylan, and (iv) normal xylan. Molecular characterization of two candidate *reduced xylan* in mucilage (*rxm*) mutants was carried out using semi-quantitative PCR and a panel of *P. ovata* genes implicated in xylan and cell wall polysaccharide biosynthesis (Phan et al., 2016). One of the mutants, *rxm1*, exhibited a reduction in the abundance of specific xylan-associated transcripts, suggesting an upstream regulator may have been compromised. These data confirm that FTMIR is a useful, non-destructive technique that can complement current screening approaches to identify putative HX mucilage mutants.

## Materials and Methods

### Plant Material and Fourier Transform Mid-Infrared (FTMIR) Spectroscopy

*Plantago ovata* seeds were mutagenized using a Co^60^ gamma source at the Australian Nuclear Science and Technology Organisation (ANSTO, Sydney, NSW, Australia). Approximately 1500 irradiated (300 Gy) *P. ovata* M1 seeds were propagated as individual lines to the M2 generation by single-seed descent. For M1 lines 1 to 315, between 3 and 15 M2 daughters were harvested individually to give ~4500 M3 seed bags for screening. M2 sister plants have a ~1:4 chance of being homozygous for somatic recessive mutations and M3 seed can therefore be screened for highly penetrant seed coat phenotypes. A total of 300 M3 lines (seed samples) were used in this study, including 160 that were pre-screened with RR stain, and 140 that were randomly selected and had not been RR-screened previously. These 300 lines represent a total of 88 M1-derived families, meaning that multiple sisters from the same family were analyzed in some cases. A WT line and two distinct mutant lines previously identified by RR staining, 42-2 and 252-7, were used as controls. Ten seeds from each line were selected from each seed bag as biological replicates for FTMIR.

Seed samples were scanned dry using a platinum diamond ATR single reflection sampling module cell mounted in a Bruker Alpha spectrometer (Bruker Optics GmbH, Ettlingen, Germany). The MIR spectra were recorded on OPUS software version 6.5 provided by Bruker Optics. Reference background spectra were recorded using no substance on an average of 32 scans, and reset every 15 seed samples. Seed spectra were recorded using individual seeds with their convex sides facing down against the diamond cell; the pressure clamp was applied to each seed for optimal sample contact. Each spectrum was obtained by taking the average of 32 scans between 4000 and 400 cm^-1^, at a resolution of 4 cm^-1^. Kimwipes^®^ were used to clean the ATR cell to prevent carry over between samples.

### FTMIR Normalization and Data Analysis

Spectra were exported from OPUS software into Unscrambler software version 7.8 (CAMO PROCESS AS, Oslo, Norway) for chemometric analysis and modified/normalized in the following order: (1) baseline offset between variable 4000 and 400 cm^-1^; (2) Savitzky–Golay (S.Golay) smoothing (parameters: averaging left/right side points = 20/20; polynomial order = 2) between variable 4000 and 400 cm^-1^; (3) baseline offset between variable 1800 and 600 cm^-1^, and defined as the “fingerprint region”; (4) definition of a new set of variables as ‘xylan characteristic frequencies,’ over 1385 ± 3, 1376 ± 3, 1365 ± 3, 1348 ± 3, 1312 ± 3, 1164 ± 3, 1134-1118, 1089 ± 3, 1070 ± 3, 1047 ± 3, 1040 ± 3, 1015-995, 986 ± 3, 897 ± 3, 856 ± 3 and 811 ± 3 cm^-1^. A preliminary PCA was performed on the “fingerprint region” with random cross validation method (10 × 30) and 12 principal components (PCs), and a xylan-specific PCA on the “xylan characteristic frequencies” with random cross validation method (10 × 30) eight PCs. The 300 tested lines were plotted according to their normalized PCA scores for PC1, PC2 and PC3.

### Ruthenium Red (RR) Staining

*Plantago ovata* seeds were placed individually in a Greiner bio-one 96-well microplate (12 seeds per line × 8 lines per plate) and imbibed in 200 μL 0.01% (w/v) RR (Sigma, 84071, Germany). Imbibition occurred at room temperature with minor agitation. The staining pattern was scored by eye at 5, 10, 20 and 30 min post-imbibition and overnight.

For high-magnification microscopy, seeds of putative mutants were imbibed for 10 min at room temperature in 0.01% (w/v) RR (Sigma, 84071, Germany) on single cavity microscopy slides under coverslips, with a total of four seeds per slide. Finer details were observed using a Zeiss Stemi 2000-C dissecting microscope and images were taken post-imbibition with an attached AxioCam ERc 5s Camera.

### Immunolabeling of Mucilage Heteroxylan (HX)

The protocol from Phan et al. (2016) was adapted for examination of HX in *P. ovata* mucilage. In brief, seeds were initially imbibed in 1X PBS for 30 min at room temperature, followed by a 60 min incubation in a 10-fold diluted primary LM11 (McCartney et al., 2005) or CCRC-M110 antibody with gentle agitation. The monoclonal CCRC-M110 antibody raised against *Phormium tenax* xylan was shown to bind *P. ovata* HX following the assay described in Pattathil et al. (2010). Samples were washed in 1X PBS (5 × 1 min) and subsequently incubated for 60 min in a 100-fold dilution of goat anti-rat IgM conjugated with DyLite 550 (Thermo Fisher, USA) with mild agitation. Samples were again washed with 1X PBS (5 × 1 min). Whole seeds were individually mounted in 1X PBS on single cavity microscopy slides and images taken using a Zeiss M2 AxioImager with Zeiss filter set 43 (excitation BP 545/25, beam splitter FT 570, emission BP 605/70) and an AxioCam Mrm black and white camera. Images were processed using ZEN 2012 software (Zeiss, Germany).

### Hot Water Mucilage Extraction and Mucilage Preparation

The method for *P. ovata* seed mucilage extraction was adapted from Balke and Diosady (2000). Thirty seeds per line were hydrated in 10 mL milliQ water and placed on a magnetic stirrer at 80°C for 90 min. The suspension of seeds and mucilage was separated by plunging the mixture through a filter sieve of fine mesh cloth in a syringe (Phan et al., 2016). Mucilage suspension samples were snap-frozen in liquid nitrogen and freeze dried for 48 h. For monosaccharide analysis, dry mucilage samples were weighed and redissolved to yield mucilage solutions with the same known concentration (2 mg/ml), and stored at -20°C.

### Monosaccharide Profiling

Monosaccharide profiles were determined using a method adapted from Burton et al. (2011) and Phan et al. (2016). Mucilage samples (400 μg/mL) were acid hydrolysed. Reaction conditions, post hydrolysis sample treatments and reverse phase high performance liquid chromatography (RP-HPLC) calibrations were carried out as described in Phan et al. (2016) with the following variations. New monosaccharide standards were prepared to enable detection of low abundance sugars in the diluted mucilage samples, and the abundance of mucilage monosaccharides was calculated as a % (w/w) i.e., the weight (mg) of monosaccharide per 100 mg dried extracted mucilage.

### Semi-Quantitative Polymerase Chain Reaction (semi-qPCR)

Integument/seed coat tissues from a minimum of 40 young developing M4 seeds (~10–12 DAP) were collected from *P. ovata* WT (3 biological replicates) and mutants (2 × M3 lines as biological replicates) and snap frozen in liquid nitrogen. RNA was extracted using the Spectrum^TM^ Plant Total RNA kit (Sigma, STRN250-1KT, USA), and cDNA synthesis was carried out using SuperScript^®^III RT (Invitrogen, 18080-044, USA) in a 96-well plate format according to the manufacturer’s instructions. Semi-qPCR reactions containing 2 μl of diluted cDNA (1/20), 5 μl of KAPA SYBR FAST qPCR mastermix (Geneworks KP-KK4602 Australia), 1 μl each of forward and reverse primers at 4 μM and 1 μl of water were carried out using a CFX384 Touch Real-Time PCR machine (Biorad, 1855485, USA). Primer pairs are shown in Supplementary Table S1. A control PoGAP gene was used as the reference for normalization. Each sample was assayed twice for every gene and the mean of the two cycle threshold values (Ct) was recorded for each gene in each plant. The averaged mean Ct’s among three WT replicates for all tested genes were used as a reference. The mean Ct for each gene of interest (GOI) in each mutant was normalized to PoGAP

$$\mathrm{Normalized mutant PoGOI Ct}=\mathrm{Mutant PoGOI Ct}\times \frac{\mathrm{avg.WT}\;\;PoGAP\,\;\;\mathrm{Ct}}{\mathrm{Mutant}\;\;PoGAP\,\;\;\mathrm{Ct}}$$

The difference in Ct numbers (n) was presented as the difference in transcript abundance Log2.

## Results

### Generation of a *P. ovata* Mutagenized Population

**FIGURE 1:**
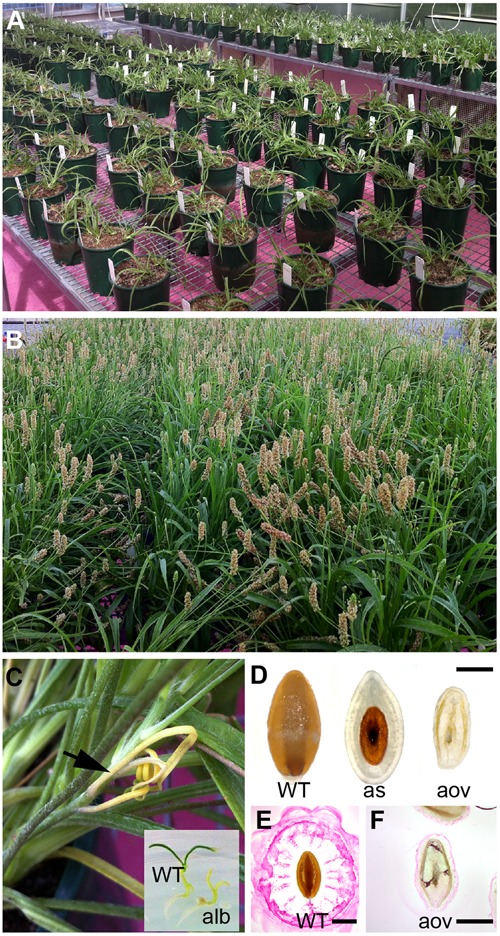
**Generation of a mutagenized *P. ovata* population for screening seed coat mucilage defects. (A)** Gamma irradiated *P. ovata* seed were germinated on petri dishes prior to being transferred into soil. **(B)** Plants were self-fertilized and seeds were collected from individual lines. **(C)** Albino sectors (see arrow) and seedlings were detected in M2 progeny consistent with successful mutagenesis. **(D)** Approximatley 30% of M2 plants displayed some form of seed or ovule lethality in the flowers. **(E,F)** Aborted ovules **(F)** showed distinctly different ruthenium red (RR) staining patterns compared to WT-like **(E)** seeds after 15 min imbibition. Bar = 1 mm. WT, wild-type; alb, albino; as, aborted seed; aov, aborted ovule.

Mature, dry *P. ovata* seeds were mutagenized using gamma irradiation. Multiple doses were tested including 50, 150, 300, 450 and 600 Gray (Gy), partially overlapping with previous irradiation studies of *Plantago* (Dodiya and Khatik, 2013). Compared to untreated controls, seedling germination was only slightly reduced for the 50 and 150Gy treatments (90%, *n* = 130 and *n* = 120) while the 600Gy treated seeds showed severely reduced (47%, *n* = 115) germination capacity. The 300Gy treated seeds showed intermediate germination efficiency (65%, *n* = 150) and were chosen for further analysis. M1 seeds were grown in large pots (**Figure 1A**), allowed to self-fertilize (**Figure 1B**) and subsequently maintained by single-line descent. Typical markers of successful mutagenesis were observed to segregate in the M2 generation including meristematic sectors and albino seedlings (**Figure 1C**), altered plant height, delayed flowering, and ovule and seed abortion (**Figures 1D–F**). M3 seed was harvested from between three and fifteen M2 sister plants, resulting in an overall population size of approximately 4500 M3 seed samples.

### Fourier Transform Mid-Infrared (FTMIR) Spectroscopy Identifies Differences in Absorbance of *P. ovata* Seed Samples

**FIGURE 2:**
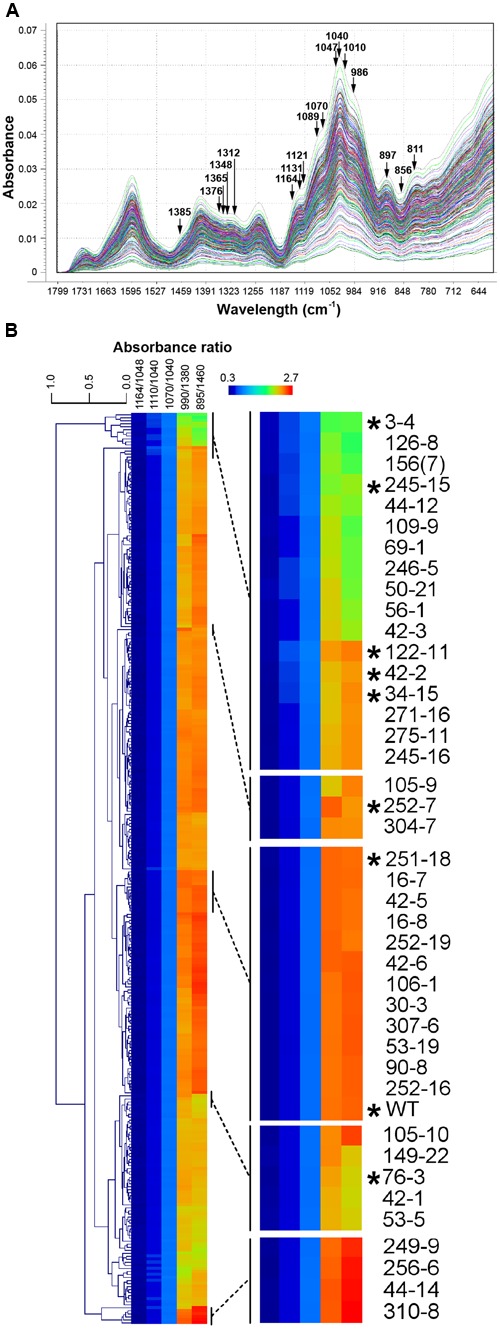
**Fourier Transform Mid Infrared (FTMIR) screening and categorization of 300 gamma-irradiated *P. ovata* lines. (A)** FTMIR spectral plots (1800 to 600 cm^-1^) of absorbance vs. wavenumbers of the 300 *P. ovata* M3 lines across the fingerprint region post data normalization. All 300 lines are represented in different colors. Wavenumbers highlighted with arrows are associated with vibrations of molecular functional groups present in xylan. Peaks with the most noticeable differences in absorbance are observed at 1376, 1365, 1312, 1131-1121, 1089-1070, 1010, 986 and 897 cm^-1^and valleys at 1348 and 856 cm^-1^. **(B)** Clustered heatmap-based comparisons of five absorbance ratios derived from **(A)**. Regions of the main plot have been expanded to show mutants (indicated with asterisks) that were analyzed further in this study.

Three hundred M3 seed samples (hereafter referred to as lines) were selected for FTMIR analysis, of which 160 had been previously screened using RR as a mucilage stain (**Figure 1E**). WT lines were included as a general control along with two mutant lines showing distinctive staining patterns, 252-7 and 42-2. Whole dry seeds were analyzed by FTMIR, and absorbance values versus wavenumbers were plotted using Unscrambler software after normalization and baseline correction. Some absorbance was detected in the lipid dominated region (3600 to 2700 cm^-1^) but absorbance predominated in the fingerprint region (1800 to 500 cm^-1^; **Figure 2A**), which contains information related to polysaccharide content. All 300 M3 lines produced a similar peak/band profile across the different regions.

Principal component analysis (Supplementary Figure S1) was carried out to distinguish the 300 lines based on the fingerprint region. Loadings (Supplementary Figure S1) of the first three principal components, PC1, PC2, and PC3 suggested separation was mainly attributed to regions from 1650 to 1550 cm^-1^, 1400 to 1300 cm^-1^ and 1150 to 800 cm^-1^, which contained a major set of wavenumbers related to xylan structure (Kaèuráková et al., 1999; Kačuráková and Wilson, 2001). When specifically examined at these wavenumbers, spectra displayed peaks and valleys with distinctive differences in absorbance values (**Figure 2A**). A semi-quantitative estimation based on five absorbance ratios including 1164/1048, 1110/1040, 1070/1040, 990/1380 and 895/1460 cm^-1^ related to arabinose substitution in AX (Kačuráková et al., 1994) was used to further interrogate the *P. ovata* population. An example of the variation in absorbance ratio across the population is shown in Supplementary Figure S2 for the 1070/1040 cm^-1^ wavelengths. Some lines were identified repeatedly in the outlier portion for more than one ratio (Supplementary Table S2). To assess variation between the lines, the five absorbance ratios for each line were compared via clustered heatmaps (**Figure 2B**). WT *P. ovata* seed showed a similar absorbance profile to multiple lines that clustered together in the middle of the heat map (**Figure 2B**). Seed from 252-7 showed a profile distinct from WT and other lines, and 42-2 showed a similar profile to uncharacterized lines including 34-15 and 122-11. The 3-4, 76-3 and 245-15 lines showed contrasting profiles at opposite ends of the heat map. This analysis enabled the identification of lines showing different absorbance ratios compared to the majority of the test lines, and to the WT control.

### Principal Component Analysis Confirms Differences Are Present between Mutant Candidates

To further define putative mutants associated with mucilage HX, a second PCA was performed based on the xylan structure-associated wavenumbers (Kačuráková et al., 1994). Separation of the 300 M3 lines was achieved as indicated by the PCA scores plot (Supplementary Figure S3). PC1 explained more than 99% of sample variance in this analysis, while PC2 and PC3 explained less than 1%. Loadings suggested separation of the lines was attributed to variable wavenumbers, in PC1, from 1047 to 900 cm^-1^, in PC2, from 1385 to 1090 cm^-1^, and in PC3, 897, 856 and 811 cm^-1^ (Supplementary Figure S4). In the PC1-PC2 score plot, the majority of the lines were found close to the central region (PC1: -0.06–0.06; PC2: -0.03–0.03), whilst fewer were detected at the periphery (Supplementary Figure S3A). The WT, 252-7 and 42-2 lines were located in the peripheral regions. A similar distribution was observed in the 3D score plot (Supplementary Figure S3B), with the majority of M3 lines located in a central core, and a smaller subset scattered in different directions in or near the PC1-PC2 plane. Two lines in particular, 122-11 and 252-7, were distinct in the 3D score plot as being distant from the PC1-PC2 plane. Irrespective of whether the specific xylan-associated wavelengths or the whole fingerprint region was used as a loading for PCA, the majority of the lines that appeared as outliers were similar.

### Correlations between Spectral Variation and Mucilage Staining

Thirty seven lines (including the 42-2 and 252-7 controls) representing 22 M1-derived families were deemed putative HX mucilage mutants based on their position in PCA plots and clustered heatmaps. RR staining was used to examine general differences in mucilage structure between *P. ovata* WT and the mutant candidates. Although RR stains acidic polysaccharides such as rhamnogalacturonan 1 (RGI), which are a minor component of *P. ovata* mucilage, previous studies in diverse *Plantago* species indicate that altered RR staining typically correlates with altered mucilage composition (Phan et al., 2016). Twelve seeds of each line were screened with RR in microtiter plates and 21 lines showed reproducible differences in overall mucilage appearance compared to WT at 10 min post-imbibition (for examples see Supplementary Figure S5A).

**FIGURE 3:**
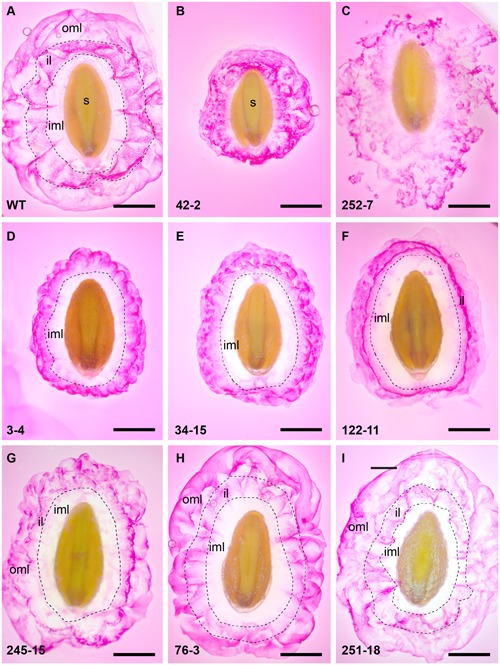
**Summary of RR staining patterns of seed from *P. ovata* WT and putative mutants.** Seeds were imbibed in 0.01% (w/v) RR stain and observed after 10 min, revealing extruded mucilage structure. **(A)** *P. ovata* WT **(B)** 42-2 **(C)** 252-7 **(D)** 3-4 **(E)** 34-15 **(F)** 122-11 **(G)** 245-15 **(H)** 76-3 and **(I)** 251-18. iml, inner mucilage layer; il, interface layer; oml, outer mucilage layer; images are shown to scale and the scale bar = 1 mm.

Additional analysis of RR-stained seeds at higher magnification revealed a variety of staining patterns. WT seed mucilage showed the typical pattern of an inner translucent layer (inner mucilage layer) surrounded by a stained interface layer containing hexagonal shapes and an intermittently stained cloud-like pink outer mucilage layer (**Figure 3A**). Mutant 42-2 showed a compact staining pattern lacking the inner translucent layer (**Figure 3B**) and 252-7 showed a patchy disorganized staining pattern that diffused rapidly into the RR solution (**Figure 3C** and Supplementary Figure S5B). Eleven lines including 3-4 (**Figure 3D**) and 34-15 (**Figure 3E**) displayed a compact RR staining pattern relative to WT at 10 min post-imbibition, either because the outer layer of mucilage was lacking or did not expand as far (Supplementary Table S3). Five lines, including 122-11 (**Figure 3F**) showed an intensely stained interface layer (**Figure 3F**), which appeared as a uniform smooth ring when observed in the microtiter plates (Supplementary Figure S5A). Line 245-15 showed a smaller mucilage ring in microtiter plate assays (Supplementary Figure S5A), but after staining on slides appeared only slightly reduced compared to WT (**Figure 3G**). Seeds from seven lines showed a staining pattern similar to WT (**Figure 3A**), including 76-3 (**Figure 3H**) and 251-18 (**Figure 3I**). Finally, nine lines appeared similar to WT in microtiter plate assays, but occasional irregular bumps of mucilage were identified after staining on slides (Supplementary Figure S6).

### Monosaccharide Compositional Analysis Reveals Altered Heteroxylan Levels in the Seed Mucilage of Several Mutant Lines

**FIGURE 4:**
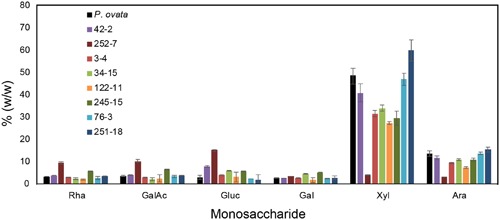
**Monosaccharide composition of mucilage from *P. ovata* WT and 8 putative mutants.** Mucilage was harvested from M4 seed samples. Monosaccharides released by acid hydrolysis were detected by HPLC and amounts are shown as % (w/w) i.e., mg/100mg of dried mucilage. Error bars show standard deviation for two biological replicates and six technical replicates.

Seed mucilage from eight putative mutants (3-4, 34-15, 42-2, 76-3, 122-11, 245-15, 251-8, and 252-7) was analyzed for monosaccharide composition (**Figure 4**). Consistent with previous studies of *P. ovata*, xylose and arabinose were the most abundant sugars in mucilage samples. Large variations in the amount of xylose and arabinose was observed between different lines; 42-2 and 76-3 showed no significant difference compared to WT, 251-18 showed an increased amount relative to WT, and the remaining lines (3-4, 34-15, 122-11, 245-15 and 252-7) showed a reduced amount of xylose and arabinose relative to WT, with a particularly large reduction in 252-7 (**Figure 4**). In most samples, rhamnose, galacturonic acid, glucose, and galactose were barely detected above background, and typically contributed less than 5% of the mucilage. The exception was 252-7, where rhamnose, galacturonic acid and glucose levels were elevated compared to WT.

### Immunolabeling Highlights Subtle Morphological Differences in Mucilage HX Deposition in the Compact Mutant Class

**FIGURE 5:**
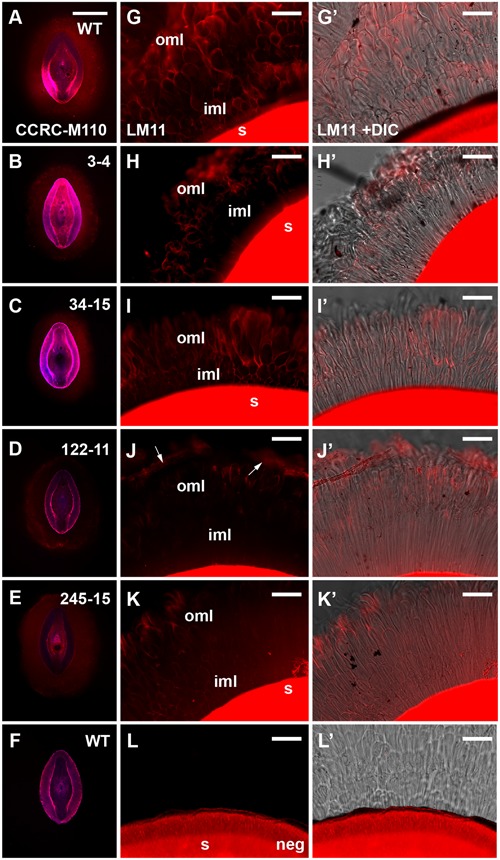
**Immunolabeling of mucilage heteroxylan (HX) by CCRC-M110 and LM11 xylan antibodies in *P. ovata* WT and four putative “compact” mutants. (A–F)** CCRC-M110, images are shown to scale. **(G–L’)** LM11. **(A)** *P. ovata* WT. **(B)** 3-4. **(C)** 34-15. **(D)** 122-11. **(E)** 245-15. **(F)** *P. ovata* WT negative control. **(G)** *P. ovata* WT. **(H)** 3-4. **(I)** 34-15. **(J)** 122-11. Arrows indicate the zones of labeling at the periphery. **(K)** 245-15. **(L)** *P. ovata* (negative control with secondary Alexa Fluor^®^550 antibody but no primary antibody). **(G’–L’)** show the overlay with the differential contrast (DIC) image. **(K)** shows auto-fluorescence originating from the seed coat. iml, inner mucilage layer; oml, outer mucilage layer. Scale bars in **A–F** = 1 mm, **G–L’** = 50 μm.

To further address the apparent reduction in xylose and arabinose content in the extruded mucilage of the compact phenotype lines (3-4, 34-15, 122-11 and 245-15), wholemount immunolabeling was conducted using the CCRC-M110 and LM11 antibodies, which bind *P. ovata* HX. Fluorescence microscopy indicated that HX epitopes were present in the seed mucilage of WT and all four putative mutants (**Figure 5**). CCRC-M110 labeling confirmed the general phenotypic differences in extrusion detected by RR (**Figures 5A–F**), and also highlighted some apparent differences in xylan distribution. For example, compared to WT seeds, mutant 122-11 displayed stronger labeling at the periphery of the mucilage and 245-15 showed diffuse labeling compared to WT in the mucilage lobes. The specific details were investigated in greater detail using the LM11 antibody for consistency with previous studies (Phan et al., 2016). In WT seeds (**Figure 5G**), lobe-like structures were observed extending out from the seed surface into the aqueous environment. Labeling was preferentially detected at the outer edge of the lobes in both the region immediately adjoining the seed (the inner transparent mucilage layer in **Figure 3A**) and the outer mucilage layers. Antibody negative controls showed no labeling in the mucilage, but confirmed strong auto-fluorescence of the residual seed tissue in the red channel (**Figure 5L**). In 3-4, 34-15 and 122-11, labeling did not extend far from the seed surface, consistent with altered mucilage organization. Line 3-4 showed reduced detection of the LM11 epitope across the mucilage, particularly in the inner mucilage layer. However, lobed structures were still labeled at the periphery (**Figure 5H**). Line 34-15 showed labeling in the inner and outer mucilage layers (**Figure 5I**), but the morphology of lobes in the outer layers was less defined than that in WT. Line 122-11 showed the most striking difference to WT, with limited labeling detected in a broad inner mucilage layer, and only diffuse labeling at the periphery (**Figure 5J**). Line 245-15 showed LM11 labeling in both inner and outer mucilage layers, but in general the labeling was not as intense as that in WT or other putative mutants (**Figure 5K**). These labeling patterns may indicate that antibodies are unable to effectively penetrate the mucilage structure or HX epitopes are missing.

### In Line 3-4, Changes Are Detected in Transcript Levels of Genes Associated with Xylan Biosynthesis

**FIGURE 6:**
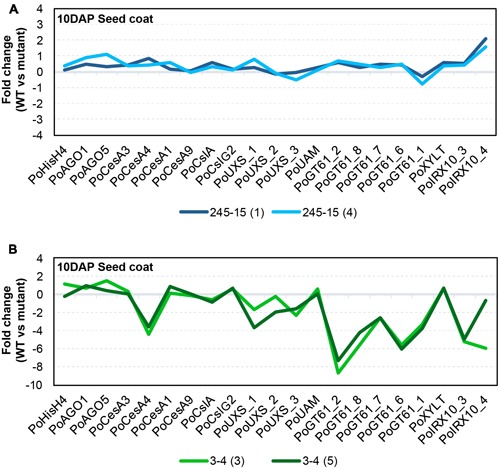
**Transcriptional behavior of selected seed-coat expressed genes in putative mutants. (A)** 245-15 (*rxm3*) **(B)** 3-4 (*rxm1*). The graphs show the transcript abundance of genes associated with cell growth and development, polysaccharide anabolism and xylan synthesis in two sister plants from each putative mutant, normalized to *P. ovata* WT. Developing seed coat samples were harvested at 10–12 days after pollination (DAP).

Gamma irradiation can lead to a diverse array of mutations including SNPs, small indels and large deletions (Morita et al., 2009), all of which have the capacity to impact gene transcript levels. Previous studies have suggested that genes encoding GT43 enzymes, such as *IRREGULAR XYLEM 9* (*IRX9*) and *IRX14* (Brown et al., 2009; Wu et al., 2009; Keppler and Showalter, 2010; Wu et al., 2010), and GT47 enzymes, such as *IRX10* (Jensen et al., 2014; Urbanowicz et al., 2014) are involved in xylan backbone synthesis, while genes encoding GT61 enzymes are involved in xylan substitution and side chain formation (Chiniquy et al., 2012). Although GT43 members appear to be present at only low levels in *P. ovata* seed coat tissues (Jensen et al., 2013), RNAseq and quantitative PCR (qPCR) have shown that GT47 and GT61 family members are highly abundant and show dynamic expression profiles during mucilage formation (Phan et al., 2016). Based on these resources, a semi-quantitative polymerase chain reaction method (semi-qPCR) was developed to interrogate transcript levels of xylan-related, general cell wall and developmental genes in the seed coat tissues of mutant candidates. The 245-15 (**Figure 6A**) and 3-4 (**Figure 6B**) lines were chosen for analysis.

RNA was extracted from seed coat tissues of WT and two independent M3 plants (i.e., M4 seeds) from each putative mutant line at 10DAP and analyzed by semi-qPCR. Most genes in 245-15 showed only subtle differences compared to WT in terms of relative transcript abundance (**Figure 6A**). The *IRX10_4* gene showed the greatest change, with approximately twofold upregulation in M4 seeds. Effects were more pronounced in 3-4 (**Figure 6B**), where a large scale reduction in transcript abundance was observed for several GT47 (*IRX10*), GT61 and UDP-Xylose Synthase (*UXS*) genes in seed coat tissues. By contrast, GT61 XYLT genes involved in β-1,2-xylosylation of N-glycans (Strasser et al., 2000; Kajiura et al., 2012) were unchanged, as were several genes involved in general polysaccharide biosynthesis including three putative cellulose synthase (*PoCesA1, 3* and *9*) and two cellulose synthase-like (*PoCslA* and *PoCslG2*) genes. A single CesA (*PoCesA4*) showed at least a fourfold reduction in transcript level. Transcript abundance of genes responsible for growth and development including *HistoneH4* (*PoHIS_H4*), *ARGONAUTE1* (*PoAGO1*) and *PoAGO5* were similar to WT. Alignment of transcript “profiles” from two sister plants for 3-4 showed a similar pattern of reduced transcript abundance in each (**Figure 6B**), suggesting that a similar defect was responsible for down-regulation of the xylan biosynthetic pathway.

## Discussion

Although much emphasis has been placed on the usefulness of *P. ovata* as a rich and accessible source of dietary fiber (Pollet et al., 2012; Cappa et al., 2013), little is known about the genetic determinants of seed mucilage formation in this species. Previous molecular studies have tended to favor myxospermous species such as *Arabidopsis* and flax where genomic sequences are available (Arsovski et al., 2009; Venglat et al., 2011; Western, 2012). However, the abundance of HX in *P. ovata* mucilage, which contrasts with pectin-rich *Arabidopsis* and flax, and its usefulness as a commercial gluten-free dietary fiber supplement make it an attractive model system for xylan research. Previous studies have detailed the use of gamma irradiation in *P. ovata* as a tool for generating genetic diversity and identifying agronomic traits that support its cultivation in India (Jain et al., 2012; Dodiya and Khatik, 2013). More recently, reverse genetic approaches have made use of the *P. ovata* system as a novel tool to identify candidate genes involved in xylan biosynthesis (Jensen et al., 2011, 2013, 2014; Phan et al., 2016). The results from this forward genetics study suggest that 300 Gy gamma-irradiation in *P. ovata* is sufficient to induce mutations that influence mucilage composition, and may provide a resource to identify novel genes and pathways involved in xylan biosynthesis. The results also support a role for FTMIR as a screening platform that might be generally applicable to other myxospermous species.

### Altered Mucilage Phenotypes Detected by FTMIR Relate to Differences in Polysaccharide Composition and Possibly Structure

A RR-based seed screen of 1500 *P. ovata* M3 lines identified approximately 100 (~7%) putative mucilage mutants, including 42-2 and 252-7. RR staining is achieved through interaction with acidic polysaccharides and has been demonstrated as a useful method to characterize seed mucilage extrusion in various myxospermous species (Fedeniuk and Biliaderis, 1994; Macquet et al., 2007; Kreitschnitz, 2009; Phan et al., 2016). However, since acidic polysaccharides such as pectins are minor components in the HX-rich *P. ovata* seed mucilage [<10% compared to >90% for HX; (Fischer et al., 2004; Guo et al., 2008; Phan et al., 2016)], we aimed to develop a complementary screening method for altered xylan composition. FTMIR was tested on 300 M3 samples (160 pre-screened with RR, 140 novel), and at least 11% of the lines showed differences in the fingerprint region and xylan-associated wavelengths of FTMIR spectra compared to the majority of lines analyzed. Of the 160 pre-screened lines, 16 were identified as putative mutants by FTMIR, of which 13 were also confirmed by RR staining. A similar frequency was identified in the 140 novel lines; 19 were identified as putative mutants by FTMIR, while in subsequent RR staining experiments, 15 were found to display altered mucilage patterns. This indicates that FTMIR can be used to support RR staining data, in addition to finding additional putative mucilage mutants (e.g., 76-3 and 251-18). The screening protocol for FTMIR is cheap and rapid, requiring no reagents and approximately 90 s to generate spectral data from each set of 10 seeds. Data integration requires familiarity with bioinformatic or chemometric software, but provides greater capacity to assess compositional variation compared to histological staining. One consideration is that putative FTMIR mutants showing no change in RR staining need to be verified using an additional method, such as monosaccharide analysis, enzyme fingerprinting, xylan-specific immunolabeling or linkage analysis. However, this is generally applicable to all FTMIR mutants, since specific structural and compositional information is difficult to precisely distinguish from the MIR spectra alone (Phan et al., 2016).

Diagnostic regions of the MIR spectra associated with AX were found to vary in the FTMIR mutants, and compared to WT, the three distinct regions stained by RR (inner transparent layer, interface layer and outer mucilage layer) often appeared to be absent or compressed. It is possible that qualitative differences in RR staining reflect modified HX release, composition and/or interactions with other polysaccharides in the different mucilage layers. Little is known about direct physical interactions between pectin and xylan in dicot species, since pectins are typically deposited in primary cell walls while xylans are more prominent in secondary cell walls (Mellerowicz et al., 2001; Rennie and Scheller, 2014). Recent studies in *Arabidopsis* suggest that small amounts of xylan may interact with both cellulose and pectin to influence the adherence of mucilage to the seed coat (Voiniciuc et al., 2015; Hu et al., 2016; Ralet et al., 2016). HX may fulfill a similar function in *P. ovata*, but addressing this requires a clearer understanding of layer organization. Guo et al. (2008) extracted different uronic and xylan components from *P. ovata* mucilage using water and hydroxide fractionation, but the spatial arrangement of these within the mucilage remains unclear. It is possible that during mucilage extrusion in *P. ovata*, RGI and HX directly interact, since both the RR staining and the LM11 labeling occurs in the cloud-like outermost lobes of mucilage surrounding WT seeds. Alternatively, HX and small amounts of cellulose in the innermost RR-negative mucilage layer (Phan et al., 2016) may contribute to RGI distribution at the periphery. Consistent with the latter, although the compact phenotype in line 3-4 originally appeared as an “outer layer” defect based on RR staining, LM11 immunolabeling suggested that changes in HX content in the inner layer may be at least partly responsible.

We hypothesized that the compact mucilage phenotypes detected in several putative mutants might be explained by an altered configuration of HX side chains, leading to reduced mucilage viscosity. Consistent with this, several lines showed a reduction in the xylose:arabinose ratio (245-15 at 2.7:1 and 34-15 at 3.1:1 compared to WT at 3.6:1), which may be indicative of altered substitution profiles. However, other compact mucilage lines (e.g., 3-4 and 122-11) showed reduced xylan labeling in specific mucilage layers, an overall reduction in xylose and arabinose content but no obvious change in the arabinose:xylose ratio. Therefore, the compact mucilage mutant analysis indicates that both HX abundance and structure in different mucilage layers may be an important physical component of mucilage extrusion. Conversely, 252-7 showed severely reduced xylose and arabinose levels, perhaps indicative of depleted xylan levels, in combination with a dispersed mucilage (**Figures 3C**, **4**, respectively). This may suggest that HX is also required to anchor mucilage to the *P. ovata* seed coat, in line with the models proposed for *Arabidopsis* mucilage xylan (Voiniciuc et al., 2015; Ralet et al., 2016). The nature of the putative 76-3 and 251-18 mutants, which contained similar or increased levels of xylose and arabinose relative to WT but distinct differences in the AX-associated wavelengths, remains elusive. These are perhaps the most interesting class of putative FTMIR mutants since they did not show an obvious difference in RR staining. It is possible they reflect subtle structural mutants, but this needs to be confirmed via linkage analysis and immunolabeling using *P. ovata* HX-specific antibodies.

### FTMIR Can Penetrate the Seed Coat to Identify Putative Mucilage Heteroxylan Mutants

Fourier Transform Mid Infrared spectroscopy utilizes intermediate wavelength IR (2.5–25 μm) and applies Fourier series mathematical treatment to spectral data (Bates, 1976). As with any IR spectroscopy, spectral data relies on the fact that bonds in functional groups in a molecule vibrate upon absorption of specific frequencies when the absorbed radiation matches the transition energy (Barth, 2007). MIR is generally used to analyze relatively pure substances, although it can detect components in simple mixtures (McDonald, 1986). In particular, analysis efficacy is subject to factors such as sample consistency, thickness and hydration. The phenotypic and compositional analyses presented here indicate that the MIR light source was able to penetrate the outer cell wall of the *P. ovata* seed epidermal cell layer and detect differences in the underlying mucilage located within the cell.

One aspect of the FTMIR methodology is the need to deconvolute IR-spectra to reveal information about specific components within a complex material. In *P. ovata* mucilage samples, HX constitutes ~90% of the mucilage dry weight. However, in the unprocessed *P. ovata* seed, the overall proportion of HX is likely to be less due to additional lipids, phenolics, proteins and other cell wall components. Despite this, the MIR spectra of the 300 lines showed strong absorbance of frequencies previously associated with varying degrees of arabinose substitution in rye xylan (Kaèuráková et al., 1999), and two mucilage mutants previously identified by RR staining were clearly distinguished from WT (Supplementary Figure S2 and **Figure 2B**). Furthermore, putative mutant lines showing similar mucilage phenotypes clustered at similar locations in PCA plots based on the whole seed data. For example, lines in the “compact” RR staining group (3-4, 34-15, 42-2) were found in a small area far to the left of the PCA plot (Supplementary Figure S3). Moreover, seed from sister plants often clustered in distinct groups (see lines from family 3 in Supplementary Figure S3), consistent with segregation of a recessive somatic-effect mutation that only impacts mucilage production in homozygous individuals. Taken together, these data indicate that the AX-associated wavelengths are able to discern differences in mucilage HX composition in unprocessed *P. ovata* seed coats, despite the complex composition of the epidermal cells.

### Multiple Genes Associated with Heteroxylan Biosynthesis Are Down-Regulated in *rxm1*

Mutant lines that are deficient in HX, or contain altered HX structure, are ideal tools to identify genes required for polysaccharide biosynthesis. Mucilage extracted from putative mutant lines 3-4, 34-15, 122-11 and 245-15 contained only ~60% of the xylose and arabinose content of the WT control. Although it is possible that some of these mutant phenotypes may relate to defects in mucilage release rather than HX synthesis, the underlying genetic lesions clearly effect the abundance of HX in the mucilage, and hereafter we refer to these putative mutants as *reduced xylan in mucilage* (*rxm*). Previous work in *P. ovata* indicated that GT family GT61 and GT47 genes are actively expressed in seed coat tissues where mucilage is produced (Jensen et al., 2013; Phan et al., 2016). GT47 genes are proposed to be involved in xylan backbone synthesis while GT61 genes influence backbone substitution.

The results presented here from semi-qPCR transcript analysis indicate that *rxm3* (245-15) plants showed only subtle changes in xylan-related gene expression while *rxm1* (3-4) plants show dramatically different transcript profiles compared to WT (**Figure 6**). While genes involved in seed growth and development, including *Histone H4* (*PoHIS_H4*) and *ARGONAUTE (PoAGO1* and *PoAGO5)*, were unchanged in *rxm1* relative to WT, a significant reduction in transcript abundance was detected for multiple GT47 (*PoIRX10_3* and *PoIRX10_4*), GT61 (*PoGT61_1*, *PoGT61_2, PoGT61_6, PoGT61_7, PoGT61_8*) and UXS genes implicated in xylan biosynthesis (Jensen et al., 2013; Phan et al., 2016). One *CELLULOSE SYNTHASE* (*CesA*) gene, *PoCesA4*, also showed significantly reduced transcript levels, while *PoCesA1*, *PoCesA3*, *PoCesA6*, *PoCslA* and *PoCslG2* were unchanged relative to WT. These results indicate that many genes involved in the xylan biosynthetic pathway are down-regulated in *rxm1*, possibly due to a genetic lesion in a shared upstream regulator. It is unlikely that the changes reflect a severe alteration in seed coat development that leads to secondary changes in HX and mucilage synthesis, since this is not apparent from the general appearance of *rxm1* seeds or transcript profiles of other seed-coat expressed genes. Further global analysis of the seed coat transcriptome for *rxm1* and *rxm3* will provide greater insight into the underlying genetic lesions and the compromised pathways.

### Differential Control of Xylan Biosynthesis in Different Mucilage Layers?

The phenotypes of *rxm1*, *rxm2* (122-11), *rxm3*, *rxm4* (34-15) and 42-2 were similar in that mucilage extrusion was decreased and some layers appeared to be diminished, absent or compressed. Previous studies by Phan et al. (2016) showed that different *IRX10* and *GT61* genes show distinct transcript accumulation patterns during seed coat development. Furthermore, distinct groups of *PoIRX10* and *PoGT61* genes seem to act in concert; for example, *PoIRX10_3* and *PoGT61_1* show similar expression profiles that differ from *PoIRX10_4* and *PoGT61_1L* (Phan et al., 2016). Although multiple xylan-related genes are downregulated in *rxm1*, *PoGT61_2* and *PoGT61_6* were particularly sensitive. The partial reduction of HX and altered mucilage appearance in the *rxm* mutants may indicate that different xylan-related genes function additively to produce an abundant HX polymer, or as independent complexes contributing distinct polymers to different layers of seed coat mucilage. The panel of mutants described here provides a resource to address the dynamics of mucilage formation in *P. ovata* and identify the underlying genetic mechanisms.

## Author Contributions

MT, RB, and DC conceived the project. MT, CM, and KN carried out screening. CM, DC, JP, and NS carried out data analysis and phenotypic assays on the mutants. MH screened antibody arrays to identify new antibodies with affinity for *P. ovata* mucilage. MT and CM wrote the manuscript with support from all authors.

## Conflict of Interest Statement

The authors declare that the research was conducted in the absence of any commercial or financial relationships that could be construed as a potential conflict of interest.
