# Supplementary material for: Dissecting the Genetic Basis for Seed Coat Mucilage Heteroxylan Biosynthesis in Plantago ovata Using Gamma Irradiation and Infrared Spectroscopy
Source: Front Plant Sci. 2017 Mar 21;8:326. doi: 10.3389/fpls.2017.00326 (PMC5359251; doi:10.3389/fpls.2017.00326)
Supplement: Supplementary file 1 [file Image_1.pdf]

Supplementary Material

Dissecting the genetic basis for seed coat mucilage heteroxylan biosynthesis in *Plantago ovata* using gamma irradiation and infrared spectroscopy

Matthew R. Tucker*, Chao Ma, , Jana Phan, Kylie Neumann, Neil J. Shirley, Michael G. Hahn, Daniel Cozzolino and Rachel A. Burton,[2]

*** Correspondence:** Matthew R Tucker: matthew.tucker@adelaide.edu.au

1 Supplementary Figures and Tables

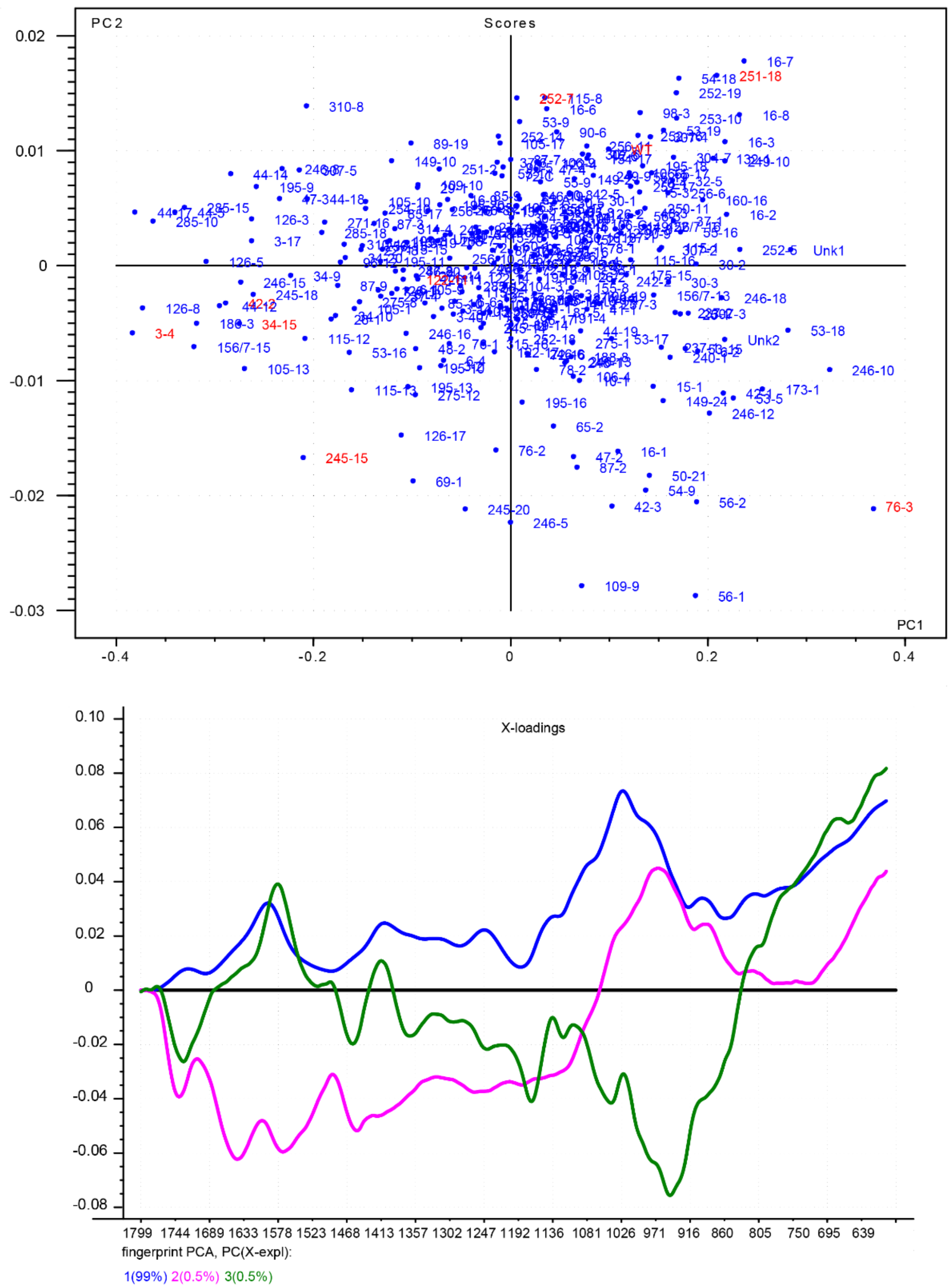

Figure S1: Principal Component Analysis of FTMIR aborbance readings derived from the fingerprint region of *P. ovata* seed samples. The upper panel shows the 2D PCA plot of normalised spectral data from 300 M3 lines including a wild-type (WT) control. Lines that were analysed in greater detail in this study are highlighted in red. The lower panel shows loadings for each principal component.

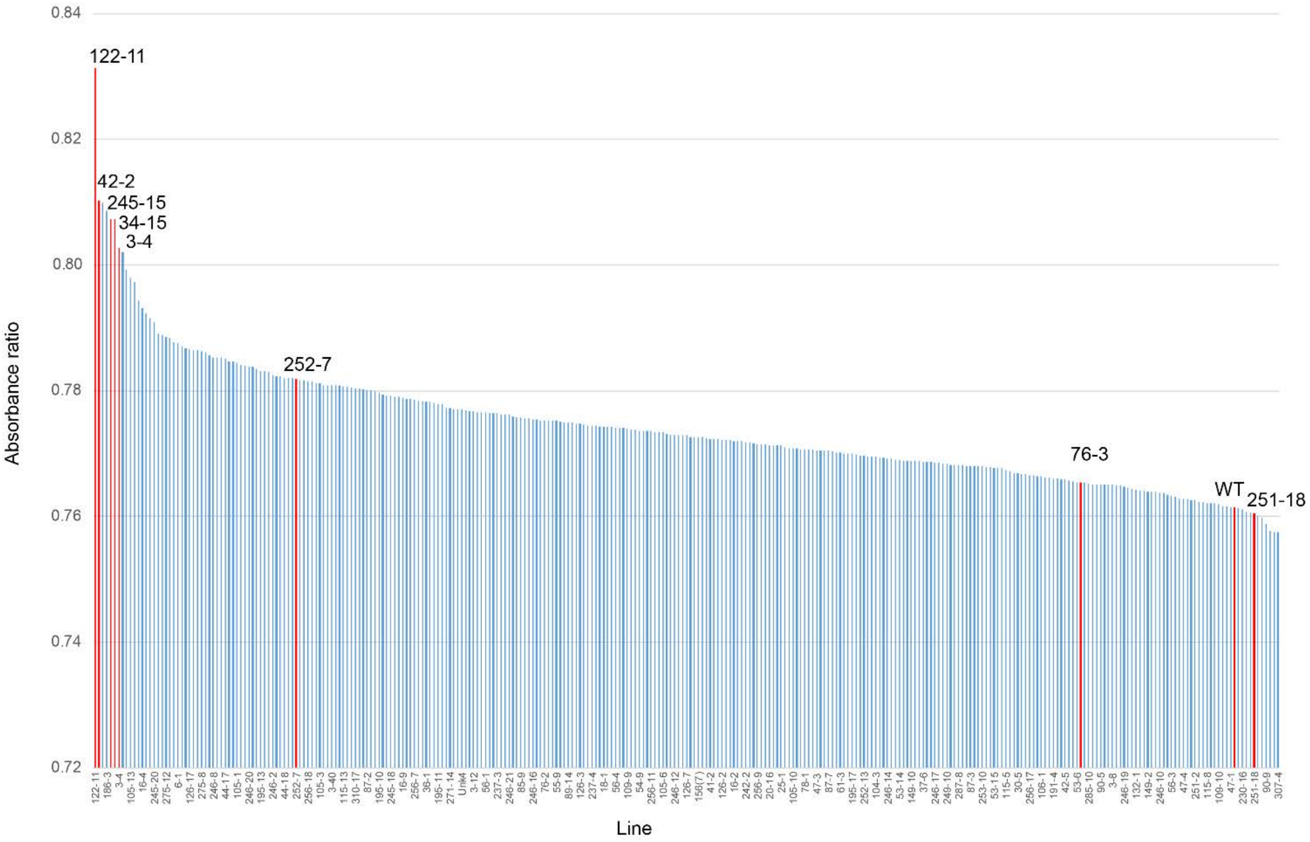

Figure S2: Absorbance ratio values of 1070cm-1/1040cm-1 from 300 *P.ovata* M3 lines and WT, sorted from largest to smallest. The same analysis was carried out for multiple xylan-associated ratios based on the normalised FTMIR data, with the aim of identifying lines showing distinct differences in heteroxylan composition/structure compared to WT. This data is shown as an example; other wavelength ratios showed distinct distribution patterns, but outlier lines were often conserved (See Table S1). The red bars indicate putative mutant candidates based on PCA analysis (see Fig S1 and S3).

A

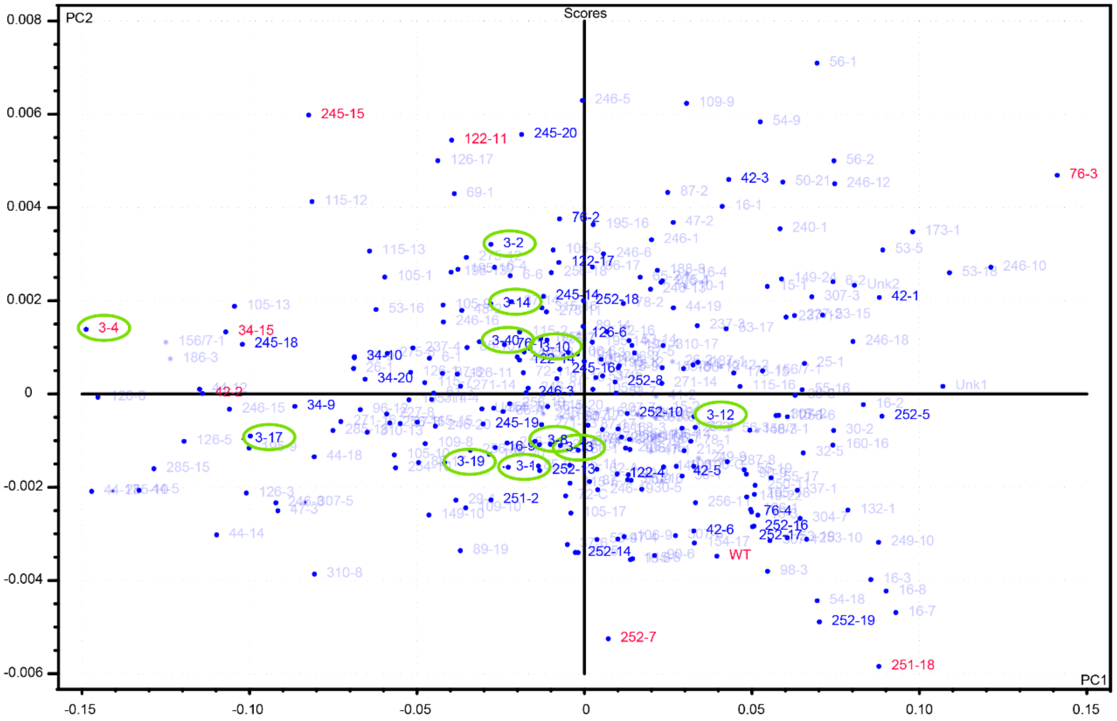

B

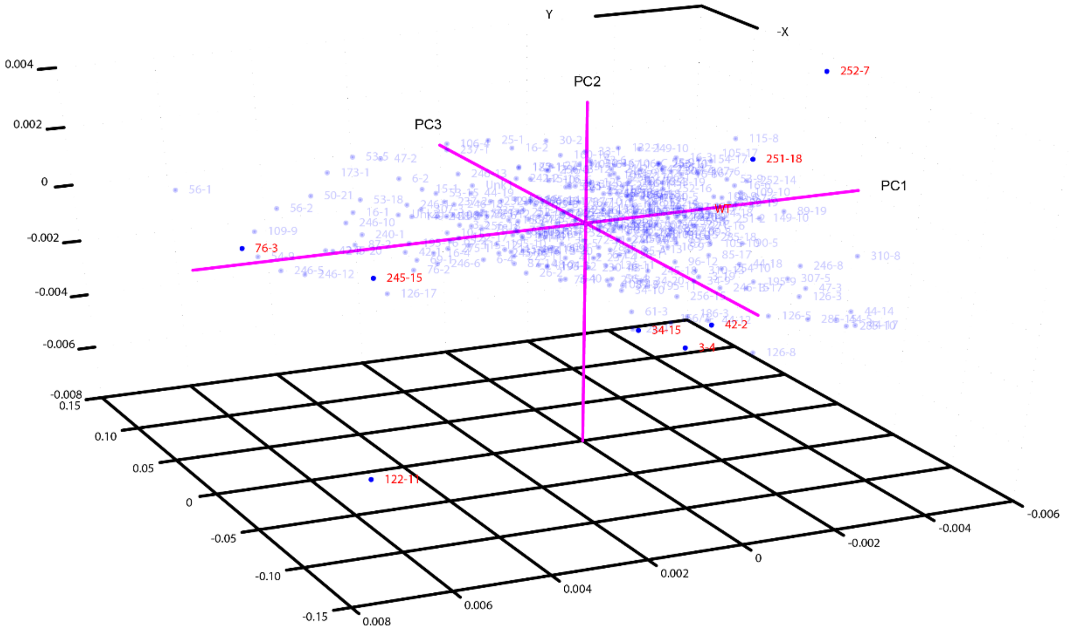

Figure S3: Principal Component Analysis of FTMIR aborbance readings derived from xylan-associated wavelengths of *P. ovata* seed samples. The upper panel shows the 2D PCA plot of normalised spectral data from 300 M3 lines including a wild-type (WT) control. Lines that were analysed in greater detail in this study are highlighted in red. Sister plants of line 3-4 are indicated with a green ring. The lower panel shows the 3D PCA plot of the same data.

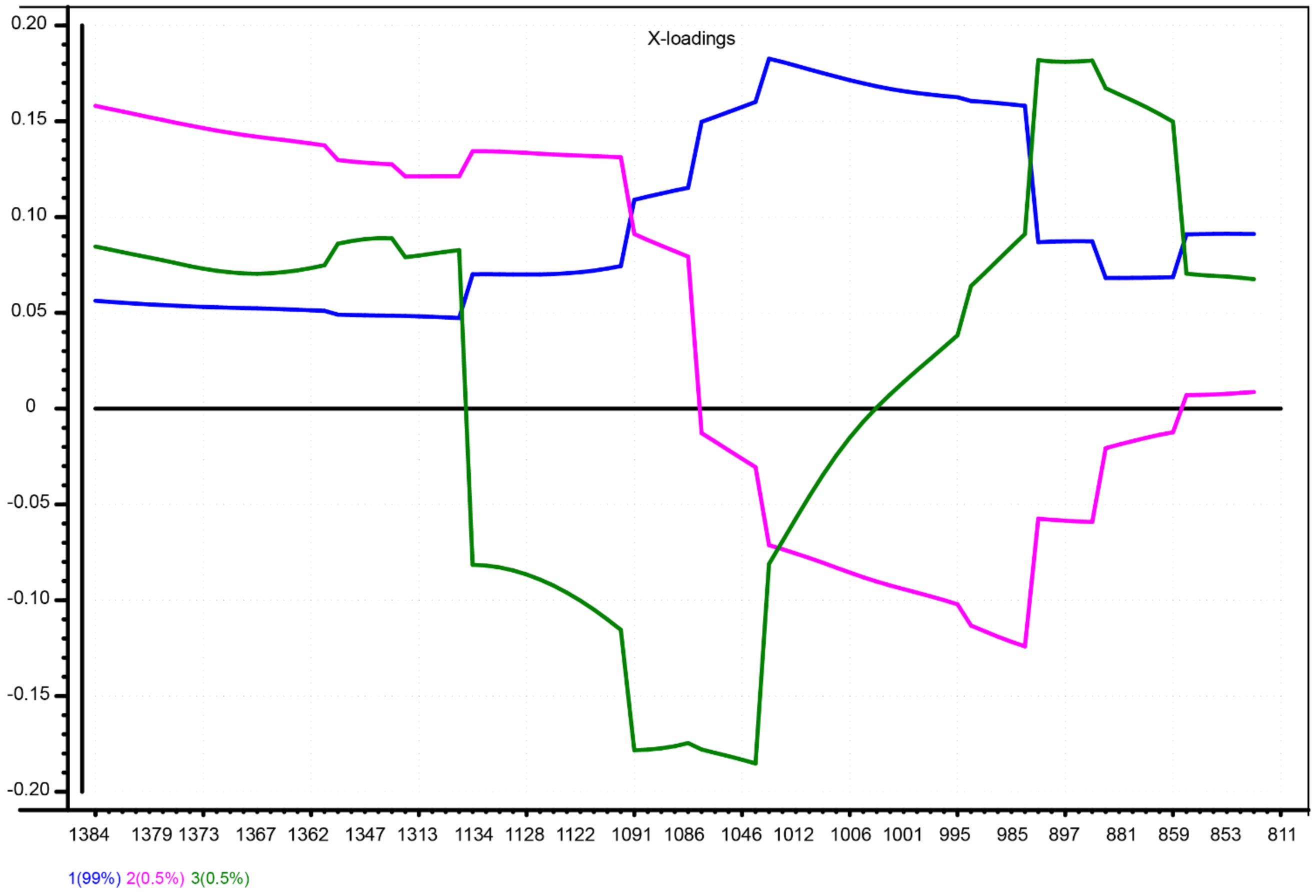

Figure S4: PCA loadings derived from xylan-associated wavelengths of *P. ovata* seed samples. The PCA plots are shown in Fig S3.

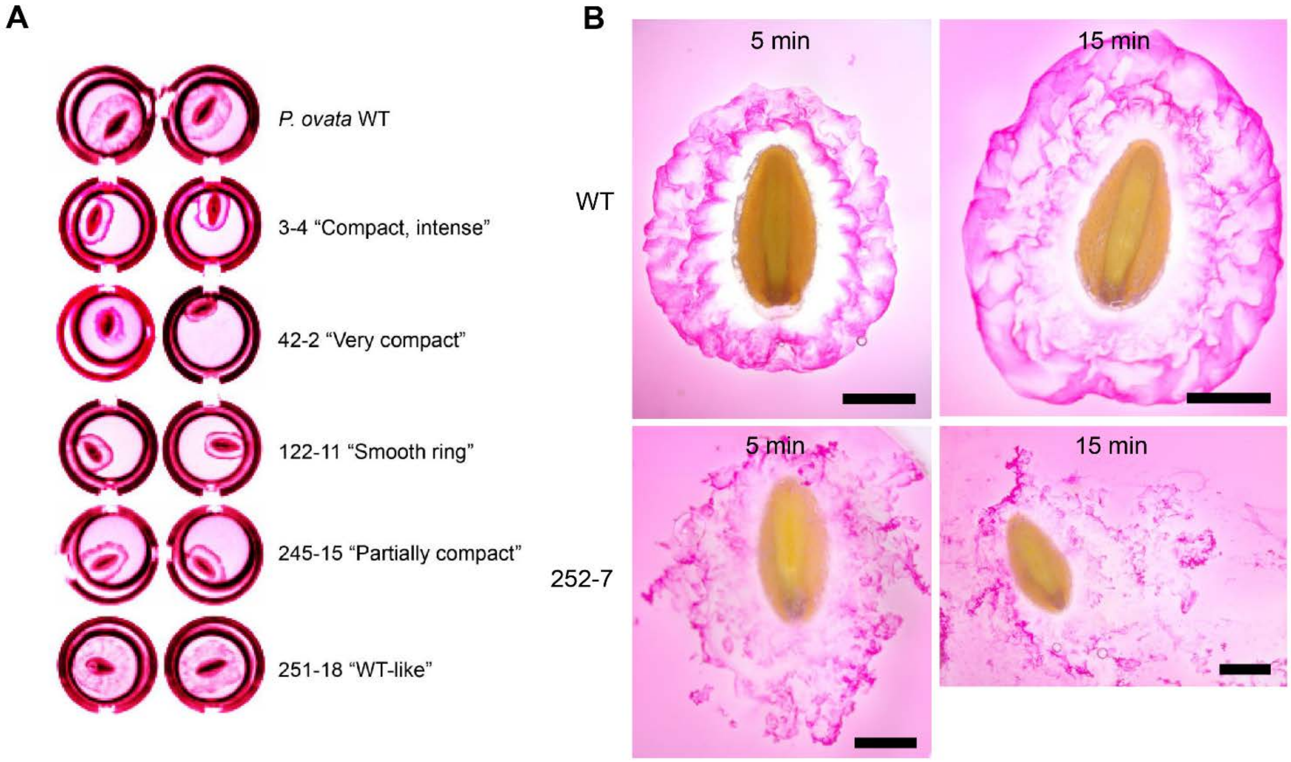

Figure S5: Ruthenium red staining of *P. ovata* seeds from different phenotypic classes. (**A**) Distinct mucilage phenotypes as they appear in microtitre plate assays. (**B**) Comparison of mucilage release and adhesion in *P. ovata* WT and 252-7 over time after RR-staining on slides. Scale bar = 1mm.

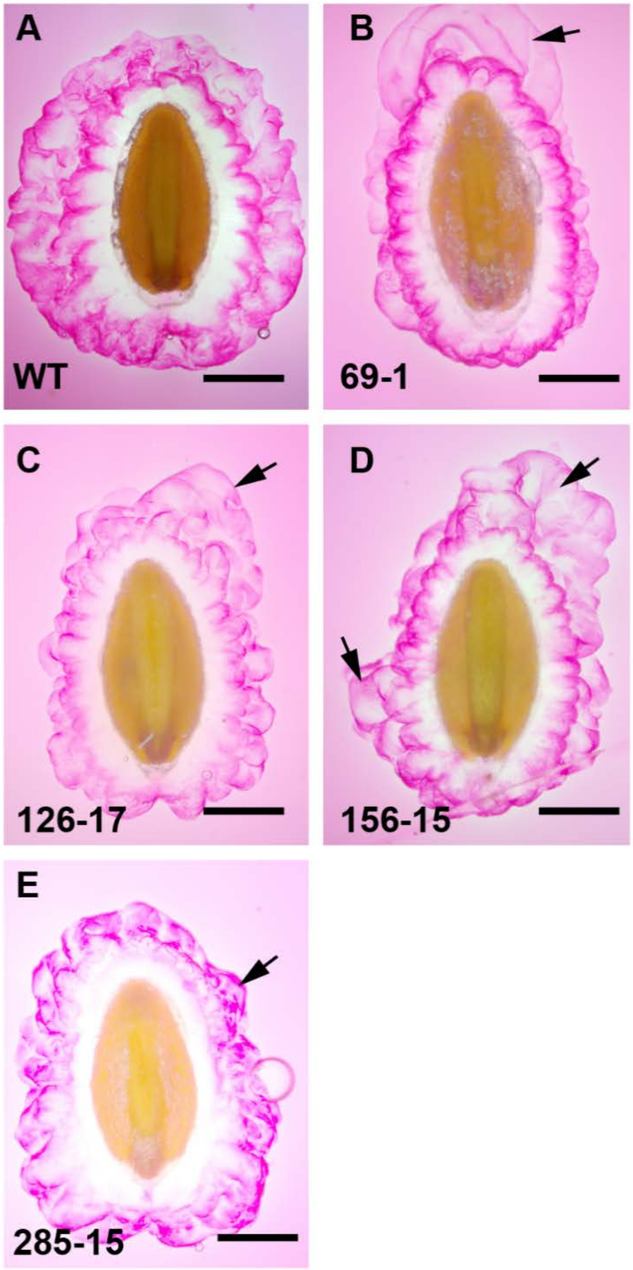

Figure S6: Ruthenium red staining of *P. ovata* seeds from M3 lines showing an "irregular bump" phenotype, as indicated by arrows. Bar = 1mm.

Table S1: Primers used for semi-quantitative PCR in this study

Name	Fwd Primer	Rev Primer	Product Size
PoGAP	TGCCTTGAGCAAGAACTTTGT	AACTGAGTGCATGTGGACGAT	100
PoHisH4	GCTGTCACCTATACCGAGCAC	TCAGCAGAAACAGAAATCCAGA	208
PoAGO1	TAGGTTTACTGGTGCCACGTC	CAACACAACACAGGCATTTGA	131
PoAGO5	GAACATACGCATTGAGGCACT	AAACACAACTCGCCAAAACAT	152
PoCesA3	GGAAATCCTAGACGACCGAGT	ACAAAAGACACGACCGACAAC	102
PoCesA4	GCAAACTGGTCCAATTCTCAA	TCAAACACAGACTTCGAGCAA	107
PoCesA1	CCTTATGAACAGTGGGGGAAT	TCATACACATCAAGGCCAACA	101
PoCesA9	TTGCTGTGGGTTCGTATCAA	CAGCAATACCCTCGGTTTGT	178
PoCslA	TTTGTTTTCCGGCTAAGGAAT	CAGCAGTAGGAAGGAGTCACG	139
PoCslG2	ATCCTGAAAGGCCATGTTTCT	GGAATAAGGACAAGCCCGTAG	223
PoUXS_1	TCTGTCGCCTTGGTGATATTT	AGCTCGCTGAGACTGTGAAAG	112
PoUXS_2	TTCTCCTGGATTTCCGAGATT	TTGATGACGGTCGTGTTGTTA	182
PoUXS_3	TACAACAGATGGGATGCAGTG	GGACTTCCTCTCATGGTGACA	140
PoUAM	CAGTTGACCCCTACTTCACCA	AACAATCCCTATCCCGTTCAC	214
PoGT61_2	CGTGCTTTCTTCTCGACTCAC	CCAACATGGTCTTTCTTCCAA	146
PoGT61_8	GGGCAAACTGTATCCAAGTGA	TCCATGGCCTTAACTAACGTG	153
PoGT61_7	TGGCTTTCAGAATGGTTTCTC	GGGAAGTACCCAATGGACAGT	139
PoGT61_6	GAATATGAAAGGGGTGCATCA	TTCACTGGCCACTTGTGAAAT	295
PoGT61_1	AAACTAATTGGGGGCAATGAC	AATGTACCTCCCGTTGGAAAC	179
PoXylT	TTCCAGGCAGAGCTGAAACTA	CACGGCTTAGCAATGAAGAAG	202
PoIRX10_3	TTCCAAATTAGCAGCCACATC	TTCCAAATTAGCAGCCACATC	275
PoIRX10_4	TATTTCCCCGATTAGGTCTGG	AAAATTTGAACGGACCTGGAT	160

Table S2: Absorbance ratio ranking of selected putative *P. ovata* mutants. The top row shows the absorbance ratios. Red coloration indicates the wild-type and internal mutant controls

Line	1164/1048	1110/1040	1070/1040	990/1380	895/1460
P. ovata	238	294	290	19	64
3-4	1	3	7	301	298
3-12	181	79	97	78	57
6-4	108	19	14	268	231
6-6	293	82	57	211	139
10-1	11	50	125	251	284
16-4	71	22	13	153	132
34-15	77	12	6	271	174
34-9	121	14	15	259	242
42-2	47	5	2	250	196
42-3	6	20	30	276	291
44-12	8	9	3	297	294
44-14	182	107	50	3	2
44-17	41	17	34	269	4
44-5	117	37	54	158	125
50-21	4	13	47	280	295
55-9	73	165	118	73	175
56-1	7	42	100	281	293
56-2	14	26	21	239	287
65-2	30	93	171	240	290
69-1	26	27	33	292	296
105-13	13	8	10	296	272
105-17	195	213	203	57	30
105-5	110	10	9	107	203
109-9	5	64	136	290	299
115-12	18	16	72	294	230
115-7	144	61	86	147	9
122-11	9	1	1	137	137
126-11	258	54	44	222	130
126-17	66	18	25	286	228
126-5	12	21	11	260	269
126-7	261	110	151	167	106
126-8	2	2	20	299	301
156-15	3	6	12	298	300
156-16	299	270	154	37	39
186-3	48	72	4	291	128
195-12	281	174	122	101	164
195-13	17	15	43	287	283
230-16	298	223	292	107	144
245-15	15	4	5	300	292
245-18	28	31	76	295	248
245-20	20	23	16	293	289
246-15	59	141	111	288	285
246-20	127	191	40	149	179
246-5	16	11	8	289	297
246-8	234	53	31	44	8
252-7	125	98	52	4	192
275-1	270	146	83	230	146
275-12	10	7	19	285	288
285-15	35	29	24	261	98
304-7	72	63	48	118	226
307-5	206	194	101	22	5
310-8	229	216	138	5	1
U-4	106	60	94	146	77

Table S3 FTMIR absorbance ratios for 37 putative *P.ovata* mucilage mutants representing 22 M1-derived families

Name	Line	RR Phenotype	1164/1048	1110/1040	1070/1040	990/1380	895/1460
rxm1	**3-4**	Compact, intense	0.435	0.610	0.803	1.818	1.840
	**16-3**	WT-like, less	0.347	0.518	0.767	2.292	2.244
	**16-7**	WT-like, less	0.337	0.513	0.761	2.423	2.413
	**16-8**	WT-like, less	0.328	0.518	0.764	2.419	2.395
	**34-9**	Compact, intense	0.345	0.557	0.792	2.187	2.216
rxm4	**34-15**	Compact, intense	0.351	0.559	0.807	2.169	2.324
	**42-2**	Very compact	0.359	0.576	0.810	2.198	2.294
	**42-3**	Very compact	0.393	0.553	0.786	2.153	1.977
	**44-5**	Smooth ring	0.346	0.543	0.782	2.278	2.373
	**44-12**	Smooth ring	0.389	0.567	0.810	1.996	1.926
	**44-14**	Smooth ring	0.341	0.532	0.782	2.456	2.632
	**44-17**	Smooth ring	0.361	0.555	0.785	2.170	2.602
	**54-9**	WT-like	0.347	0.542	0.774	2.194	2.141
	**54-18**	WT-like	0.336	0.515	0.765	2.456	2.511
	**56-1**	WT-like	0.390	0.542	0.777	2.128	1.940
	**56-2**	WT-like	0.378	0.548	0.788	2.208	2.056
	**69-1**	WT-like, bumpy	0.370	0.546	0.785	2.048	1.880
	**76-3**	WT-like	0.347	0.526	0.765	2.270	2.117
	**105-13**	Compact	0.379	0.567	0.798	2.012	2.129
	**109-9**	WT-like	0.398	0.539	0.774	2.056	1.834
	**115-12**	Compact	0.377	0.556	0.780	2.027	2.241
rxm2	**122-11**	Smooth ring	0.387	0.654	0.831	2.293	2.363
	**126-5**	WT-like, bumpy	0.386	0.553	0.797	2.187	2.142
	**126-8**	WT-like, bumpy	0.423	0.620	0.789	1.916	1.762
	**126-17**	WT-like, bumpy	0.354	0.555	0.787	2.109	2.245
	**156-15**	WT-like, bumpy	0.422	0.571	0.794	1.950	1.818
rxm3	**245-15**	Partially compact	0.378	0.582	0.807	1.913	1.966
	**245-18**	Partially compact	0.368	0.545	0.779	2.016	2.208
	**245-20**	Partially compact	0.373	0.551	0.791	2.043	2.043
	**246-5**	Compact, slow	0.378	0.562	0.802	2.071	1.873
	**246-8**	Compact, slow	0.336	0.540	0.785	2.369	2.553
	**246-15**	Compact, slow	0.356	0.528	0.776	2.078	2.074
	**251-18**	WT-like	0.354	0.519	0.760	2.426	2.400

**252-7**	Dispersed	0.345	0.533	0.782	2.445	2.298
**285-15**	WT-like, bumpy	0.364	0.546	0.787	2.185	2.399
**307-5**	Compact	0.338	0.522	0.776	2.391	2.589
**310-8**	Compact	0.336	0.520	0.774	2.448	2.712
**WT**	WT-like	0.335	0.509	0.761	2.393	2.433

RR- ruthenium red stain after 10 min imbibition, **WT** - wild-type, **compact** – RR-stained mucilage did not extend as far as WT, **intense** – appeared more heavily stained at the periphery of the mucilage compared to WT, **WT-like** – similar in appearance to WT, **WT-like, less** – similar to WT in plates and slides, but mucilage did not stain as intensely, **partially compact** – appeared compact in plates but only slightly reduced compared to WT on slides, **smooth ring** – the outer layer of mucilage stained in a perfectly smooth ring, **bumpy** – similar to WT but sporadic bumps appeared at the periphery where mucilage appears to spread faster, **dispersed** – appears punctate and disconnected from the seed, ***rxm*** – putative *reduced xylan in mucilage* mutant.
